# Effects of Mongolian Warm Acupuncture on iNOS/NO and Inflammatory Cytokines in the Hippocampus of Chronic Fatigue Rats

**DOI:** 10.3389/fnint.2019.00078

**Published:** 2020-01-31

**Authors:** Ling Shui, Ru-Na Yi, Yong-Jie Wu, Shu-Mei Bai, Qin Si, A-gula Bo, Ge-Rile Wuyun, Leng-Ge Si, Ying-Song Chen, Jun Lu

**Affiliations:** ^1^College of Traditional Mongolia Medicine, Inner Mongolia Medical University, Hohhot, China; ^2^School of Acupuncture-Moxibustion and Tui Na, Beijing University of Chinese Medicine, Beijing, China

**Keywords:** exhaustive exercise, cytokines, iNOS/NO signaling pathway, warm acupuncture, inflammation

## Abstract

The inducible nitric oxide synthase/nitric oxide (iNOS/NO) signaling pathway and inflammatory cytokines play important roles in the pathogenesis of exercise-induced fatigue. Studies have found that Mongolian warm acupuncture (WA) could alleviate exercise-induced fatigue. However, the exact mechanisms underlying its effects remain unclear. In the present study, we investigated the effects of Mongolian WA on iNOS/NO signaling pathway and proinflammatory cytokines in a chronic exhaustive swimming-induced fatigue rat model. Animals were randomly divided into Control group, Ctrl + WA group, Model group, and Model + WA group. The body weight, exhaustive swimming time test, and Morris water maze test were performed before and after the chronic exhaustive swimming. The serum levels of interleukin-1β (IL-1β), interleukin-6 (IL-6), tumor necrosis factor-α (TNF-α), interferon-γ (IFN-γ), and iNOS were detected by enzyme linked immunosorbent assay (ELISA). The mRNA expressions of IL-1β, IL-6, TNF-α, IFN-γ, and iNOS in the hippocampus were measured by real-time polymerase chain reaction (RT-PCR). Moreover, the protein expression of iNOS in the hippocampus was measured by western blot, and the NO productions in the serum and hippocampus were detected by Griess reaction system. Chronic exhaustive exercise significantly reduced the body weight and exhaustive swimming time, and induced impairment in learning and memory, and which were reversed by WA treatment. Chronic exhaustive exercise also increased the expressions of iNOS and proinflammatory cytokines, while WA treatment significantly decreased the level of iNOS and proinflammatory cytokines. However, chronic exhaustive exercise did not affect the NO production. These findings demonstrated that WA could alleviate the chronic exhaustive swimming-induced fatigue and improve the learning and memory ability, and the actions might be related to the reduction of inflammatory response and iNOS expression.

## Introduction

The aims of training in sport are to improve and optimize the performance. When the daily strength, duration, and workload of exercise are adequate, positive physiological adaptations occur in muscle and other tissues, and physical performance is generally enhanced (Li et al., 2019). However, when excessive and prolonged training stresses are applied, concurrent with other stressors and insufficient recovery, performance decrements and chronic maladaptations could occur (Meeusen et al., 2007). That, in turn, could lead to motor fatigue and overtraining (Owen et al., 2018). Chronic exhaustive exercise might cause mechanical injury of muscles and activates macrophages, which results in increased synthesis and secretion of proinflammatory cytokines, such as interleukin-1β (IL-1β), interleukin-6 (IL-6), and tumor necrosis factor-α (TNF-α). These cytokines also stimulate macrophages to secrete prostaglandins causing muscle soreness, and lead to exercise-induced fatigue (Pawlik et al., 2016; Kawamura et al., 2018; Yang et al., 2019). In addition, the hippocampus is an important brain region that is vulnerable to stress and is involved in the exercise-induced fatigue (Zhu et al., 2017). The exhaustive exercise stress could induce neuroinflammatory response in the hippocampus and cause brain damage, which impairs learning and memory (Schwarz et al., 2018; Morgan et al., 2019).

Nitric oxide (NO) is an important messenger molecule and effector molecule in the body and is closely related to pathological and physiological processes of the nervous system (Stoyanova and Lazarov, 2005). For example, NO in the hippocampus participates in the learning and memory process as a neurotransmitter (Mosher et al., 2016; Andrade Próspero et al., 2018; Berenyiova et al., 2018). The overtraining syndrome after chronic exhaustive exercise is related to the expression of NO and NO synthase (NOS; Lee et al., 2012). There are three types of NOS: endothelial NOS (eNOS), neuronal NOS (nNOS), and inducible NOS (iNOS). The iNOS is the major NOS isoform in the brain (Sun et al., 2019).

The Mongolian warm acupuncture (WA) is a traditional therapy of Mongolian medicine, which uses silver needles and gives acupuncture and warm stimulation at specified points (Bo et al., 2017). Studies have shown that Mongolian WA can regulate the neuroendocrine and immune system (Huo, 2006; Agula and Chen, 2009; Chen et al., 2010). Previously, we have found that Mongolian WA could relieve exercise-induced fatigue (Chen et al., 2008), but the exact mechanism remains unclear.

Therefore, in this study, we further investigated the underlying mechanism. We used the chronic exhaustive swimming model to investigate the effects of Mongolian WA on inducible nitric oxide synthase/nitric oxide (iNOS/NO) signaling pathway and proinflammatory cytokines. The body weight, exhaustive swimming time test, and Morris water maze test were performed before and after the chronic exhaustive swimming. The levels of proinflammatory cytokines (IL-1β, IL-6, TNF-α, and IFN-γ), iNOS, and NO were also observed in fatigue rats. Our results showed a potential inflammatory damage and increased expression of iNOS in the brain, which might be involved in the pathogenesis of fatigue induced by chronic exhaustive exercise. WA could increase the body weight and exhaustive swimming time and improve the memory ability in exercise-induced fatigue rats, which might be mediated by inhibition of the proinflammatory cytokines and iNOS in the brain region.

## Materials and Methods

### Animals

Forty male Sprague–Dawley (SD) rats, weighing 160–180 g, were provided by Beijing Vital River Laboratories Animal Technology Company Limited. The experimental animal license number is SCXK (Beijing) 2016-0011. The cages were kept in a room with controlled light (12:12 h light/dark cycle, light on/off, 7:00 AM/7:00 PM) and temperature (22 ± 22°C). The rats were given adaptive feeding for 7 days before the experiment. All the animal experiments were approved by the Institutional Animal Care and Use Committee of Affiliated Hospital of Inner Mongolia Medical University.

### Groups and Treatment

The 40 male SD rats were randomly assigned to the Control group, Ctrl + WA group, Model group, and Model + WA group. The Control group was given conventional feeding without any intervention; the Ctrl + WA group was given conventional feeding and received WA treatment once every other day during the 21-day period; the Model group was given exhaustive swimming once a day for 21 days; the Model + WA group was given exhaustive swimming once a day for 21 days and received WA intervention once every other day during the 21-day period.

### Chronic Exercise-Induced Fatigue Rat Model

The rat model was established according to our previous study (Chen et al., 2008; Lu et al., 2012). Briefly, the rats were given adaptive swimming [water temperature of (30 ± 2)°C] for 20 min in a white plastic bucket (diameter of 50 cm, height of 70 cm), once a day for 3 days. At the fourth day, the rats began to swim with loaded weight (5% of the body weight) for 21 days. The exhaustive swimming time was recorded when the rats could not return to the surface 10 s after entering the water, and then the rats were quickly picked up.

### Mongolian WA Treatment

The acupoints Dinghui (center of the parietal bone) and Xinxue (center of the inferior fovea of the seventh thoracic vertebra) were selected according to a previous study (Chen et al., 2016). The silver needle (diameter 0.5 mm, length 2 cm) was connected to the Mongolian medical MLY-I electrothermal acupuncture apparatus (Mongolian Medical College of Inner Mongolia Medical College), and the temperature was set at 40°C. The rats were given WA treatment for 15 min once every other day during the 21-day period. The Model + WA rats received WA 1 h after the end of the swim each time.

### Body Weight

The body weight of the rats was measured before and at the end of the 21-day chronic exhaustive swimming training period.

### Exhaustive Swimming Time

The exhaustive swimming time test was performed before and at the end of the 21-day chronic training period. The exhaustive swimming time was measured from the moment when the rats were dropped into water until they were completely exhausted, which was calculated by failing to rise to the surface to breathe within 10 continuous seconds.

### Morris Water Maze

The Morris water maze was used to measure spatial learning and memory abilities (Vouros et al., 2018). A milky white, circular water pool (diameter of 120 cm, height of 50 cm, platform diameter of 10 cm, height of 22 cm) was filled to a depth of 20 cm with water kept at (25 ± 1°C). The water pool was divided into four quadrants, and a cylindrical platform was placed in the center of the target quadrant such that it was submerged 2 cm beneath the surface. On the 16th day of WA treatment, 1 h after the end of treatment, each rat was subjected to one daily four-place navigation trial (six consecutive days). The average speed and the time for finding the platform (escape latency) were recorded (within 120 s). If a rat failed to reach the platform within 120 s, it was guided to the platform and permitted to stay there for 10 s, and the escape latency was recorded as 120 s. The long escape latencies represented weak learning ability. On day 22, the platform was removed and the rats were released in the quadrant opposite the quadrant where the platform had been. The number of times the rats crossed the platform that had been previously located within 60 s was recorded. A low frequency of crossing the former platform quadrant was used to indicate weak memory ability. All data were automatically recorded by a computerized video system (Chengdu Techman Software Company Limited, Chengdu, China).

### Sample Collection

Twenty-four hours after the last behavioral tests, the rats were sacrificed. The blood was taken and was centrifuged at 4,000 r/min (4°C) for 15 min, and then the supernatant was collected. The hippocampus was quickly removed and stored in −80 °C for further analysis.

### Enzyme-Linked Immunosorbent Assay (ELISA)

The contents of IL-1β, IL-6, TNF-α, IFN-γ, and iNOS in the serum were measured using commercially available ELISA kits (MultiSciences Biotech, China) according to the manufacturer’s instructions. The samples or standards were read on an ELISA plate reader at a wavelength of 450 nm.

### RT-PCR

The mRNA expressions of IL-1β, IL-6, TNF-α, IFN-γ, and iNOS in the hippocampus were measured by real-time polymerase chain reaction (RT-PCR). Briefly, the total RNA from hippocampus was obtained by the Trizol method. RNA was reverse-transcribed to cDNA using the Taqman Reverse Transcription Reagents (Applied Biosystems, Waltham, MA, USA). RT-PCR analyses were performed in 96-well plates using the ABI PRISM 7700 Sequence Detection System instrument and software. PCR was performed with the SYBR Green PCR Master Mix (Applied Biosystems, USA) according to the manufacturer’s protocol, using the following oligonucleotide primers—IL-1β: forward, 5′-GAGTCTGCACAGTTCCCCAA-3′; reverse, 5′-TGTCCCGACCATTGCTGTTT-3′(88bp); IL-6: forward, 5′-CACTTCACAAGTCGGAGGCT-3′; reverse, 5′-TCTGACA GTGCATCATCGCT-3′(114bp); TNF-α: forward, 5′-CGTC AGCCGATTTGCCATTT-3′; reverse, 5′-CTCCCTCAGGGGTGTCCTTA-3′(89bp);IFN-γ: forward, 5′-CATCGCCAAGTTCGAGGTGA-3′; reverse, 5′-TCTGGTGACAGCTGGTGAATC-3′ (87bp); iNOS: forward,5′-CACAGAGGGCTCAAAGGAGG-3′; reverse, 5′- AAAGTGGTAGCCACATCCCG-3′ (281 bp) and β-actin: forward, 5′-ACGTTGACATCC GTAAAGAC-3′; reverse, 5′-GGACTCATCGTACTCCTG CT-3′(81 bp). The basic protocol for RT-PCR was an initial incubation at 95°C for 5 min, followed by 45 cycles of 95°C for 30 s, 56°C for 30 s, 72°C for 1 min, and finally cooling to 40°C. All samples were run in triplicate and the relative expression values were normalized with a β-actin value.

To quantify the results obtained by RT-PCR, plasmids containing cDNA were used as the standard. The interest cDNA was amplified by RT-PCR using the same premiers as for RT-PCR. The PCR products were cloned into pGEM-T easy vector (Invitrogen) and confirmed by sequencing. The purified recombinant plasmid DNA was quantified by a UV spectrophotometer and then serially diluted in double-distilled water as the standard for numerical quantification. The PCR products were sequenced to verify the analytical specificity. Melting curve was analyzed after PCR amplification.

### Western Blot

The iNOS expression in the hippocampus was measured by Western blot. Briefly, the hippocampus was added into 100-μl lysate [50 mM Tris-HCl (pH 7.4), 150 mM NaCl, 1% np-40, 0.1% sodium dodecylsulfate (SDS)], shaken violently for 2 min, incubated on ice for 20 min, and then centrifuged at 13,000 rpm (4°C) for 20 min. Taking the supernatant and the protein content was determined according to the instructions of the BCA protein quantitative kit. Then, aliquots of 50-μg homogenate protein were run on 10% (w/v) SDS-polyacrylamide gel electrophoresis (PAGE) gels. Proteins were subsequently transferred to nitrocellulose membranes, which were blocked by incubation with 3% (w/v) fat dry milk in PBS for 1 h at room temperature, with shaking. Thereafter, the membranes were incubated overnight at 4°C with rabbit polyclonal anti-iNOS or rabbit polyclonal anti-actin (Santa Cruz, Biotechnology, Santa Cruz, CA, USA). The primary antibody was used at dilutions from 1:500 to 1:1,000. The blots were washed four times with Tris-buffered saline Tween-20 (TBST) and subsequently incubated for 2 h at room temperature in Tris-buffered saline with horseradish peroxidase-conjugated anti-rabbit IgG antibody (1:5,000; Pierce, Biotechnology, Waltham, MA, USA). Gel Image System ver. 4.00 software was used to scan the image for gray analysis. Results were calculated as the relative ratio of the specific band compared with actin.

### Measurement of NO Level

NO levels in serum and hippocampus were determined spectrophotometrically, based on the reduction of NO_3_^−^ to NO_2_^−^ by VaCI_3._ The NO level was measured by the Griess reaction (Nanjing Jiancheng, China). Briefly, equal volumes of Griess Reagent I and Griess Reagent II were added, and the absorbance at 540 nm was detected by a microplate reader. The NO content was calculated from a nitrite standard curve.

### Statistical Analysis

All data were expressed as mean ± standard deviation ($\bar{x}$ ± SD). Statistical analyses were performed using the SPSS 22.0 software. One-way ANOVA was used for comparison among groups, and *t*-test was used for comparison between two groups. For tests of significance, a *P*-value of less than 0.05 was considered to be significant.

## Results

### Effect of WA on Weight

**Figure 1:**
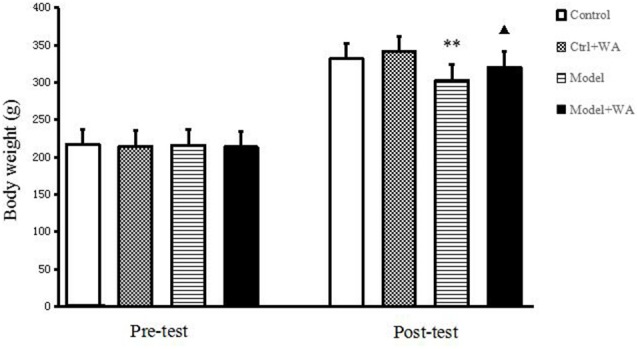
Effect of warm acupuncture (WA) on the body weight. The data are presented as the means ± SD, *n* = 10 for each group. **P < 0.01 as compared to the control group; ^▴^*P* < 0.05 as compared with the model group.

Before the chronic training experiment, the body weight of the rats was not significantly different among groups (*P* > 0.05). On the last day of the chronic training experiment, compared with the Control group, the body weight of the Model group was significantly decreased (*P* < 0.01). Treatment with WA after exhaustive swimming could significantly increase the body weight as compared to the Model group (*P* < 0.05); the results are shown in Figure 1.

### Effect of WA on Exhaustive Swimming Time

**Figure 2:**
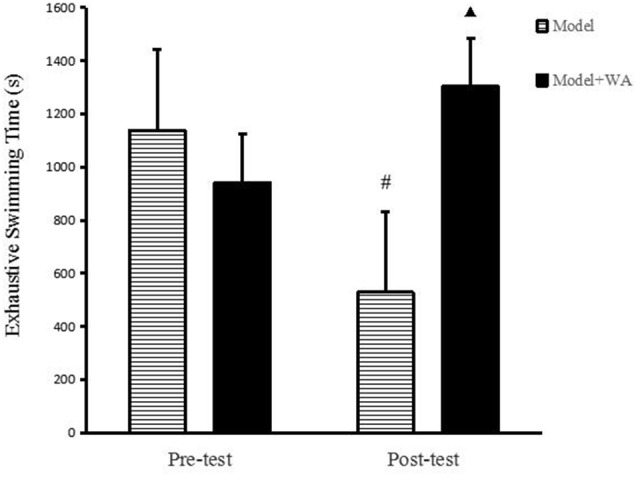
Effect of WA on exhaustive swimming time. The data are presented as the means ± SD, *n* = 10 for each group. ^▴^*P* < 0.05 as compared with the pos*t*-test Model group; ^#^*P* < 0.05 as compared to the pre-test Model group.

As shown in Figure 2, there was no significant difference on the exhaustive swimming time between the Model group and the Model + WA group (*P* > 0.05). On the other hand, the exhaustive swimming time was significantly increased in the Model + WA group compared with the Model group after 3 weeks of chronic exhaustive swimming (*P* < 0.05). In addition, the exhaustive swimming time in the Model group after the chronic training was significantly lower than that before the chronic training period (baseline, *P* < 0.05).

### Effect of WA on Morris Water Maze Test

**Figure 3:**
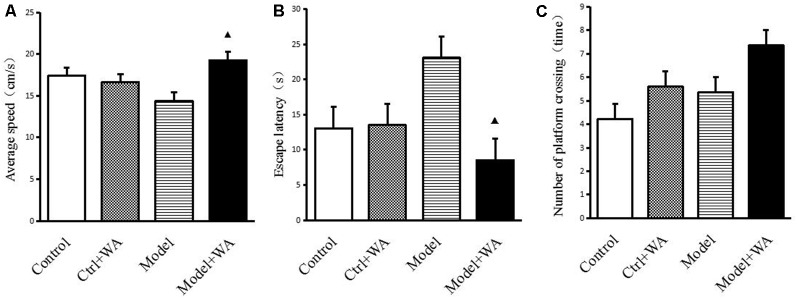
Effect of WA on the Morris water maze test. **(A)** Average speed. **(B)** Escape latency. **(C)** Number of platform crossing. The data are presented as means ± SD, *n* = 10 for each group. ^▴^*P* < 0.05 as compared to the Model group.

The average speed, the escape latency, and the number of crossing platforms were observed in the Morris water maze, as shown in Figure 3. The chronic swimming slightly decreased the average speed and mildly increased the escape latency and the number of crossing platforms, but the differences were not significant (all *P* > 0.05). Compared with the Model group, WA treatment significantly increased the average speed and decreased the escape latency (both *P* < 0.05). Although WA could slightly increase the number of crossing platforms, the difference was not significant (*P* > 0.05).

### Effect of WA on Serum Levels of IL-1β, IL-6, TNF-α, IFN-γ, and iNOS

**Figure 4:**
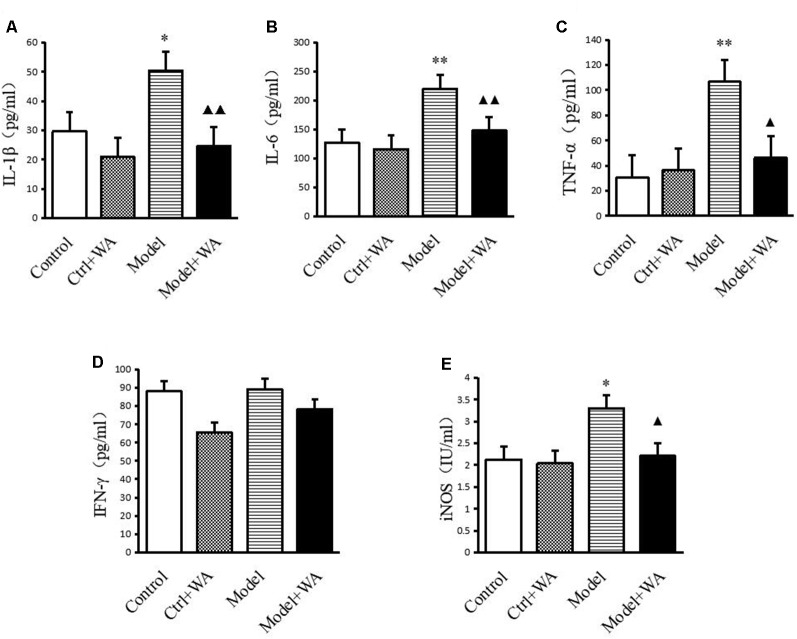
Effect of WA on serum levels of interleukin-1β (IL-1β), interleukin-6 (IL-6), tumor necrosis factor-α (TNF-α), IFN-γ, and inducible nitric oxide synthase (iNOS; **A)** IL-1β. **(B)** IL-6. **(C)** TNF-α. **(D)** IFN-γ. **(E)** iNOS. The data are presented as the means ± SD, *n* = 10 for each group. *P < 0.05, **P < 0.01 as compared to the control group; ^▴^*P* < 0.05, ^▴▴^*P* < 0.01 as compared to the Model group.

As shown in Figure 4, chronic exhaustive swimming significantly increased the levels of IL-1β, IL-6, TNF-α, and iNOS in serum (*P* < 0.05, *P* < 0.01, *P* < 0.01, and *P* < 0.05, respectively). WA treatment markedly decreased the chronic exhaustive swimming induced increase in the expressions of IL-1β, IL-6, TNF-α, and iNOS (*P* < 0.01, *P* < 0.01, *P* < 0.05, and *P* < 0.05, respectively). However, there were no statistically significant differences on the IFN-γ level among the four groups (*P* > 0.05).

### Effect of WA on mRNA Expressions of IL-1β, IL-6, TNF-α, IFN-γ, and iNOS in the Hippocampus

**Figure 5:**
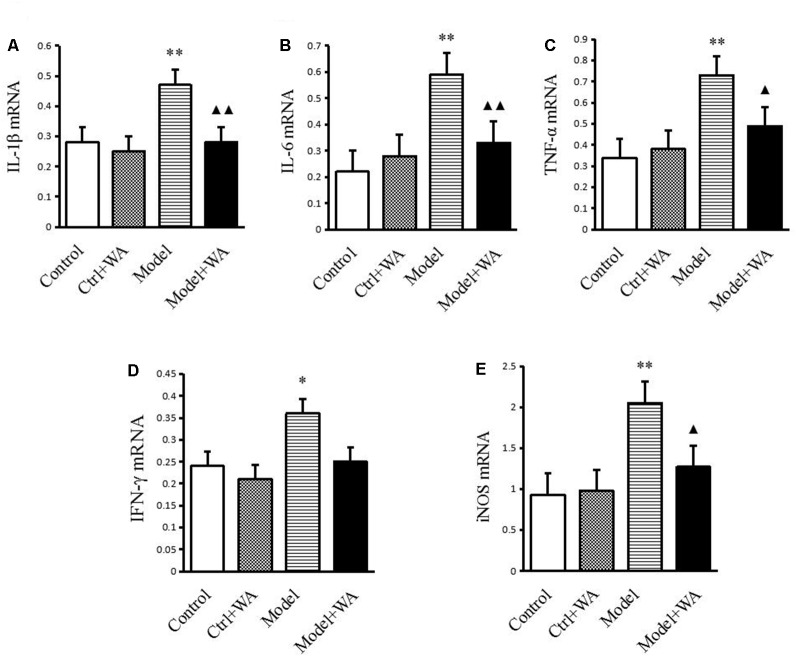
Effect of WA on mRNA expressions of IL-1β, IL-6, TNF-α, IFN-γ, and iNOS in the hippocampus. **(A)** IL-1β. **(B)** IL-6. **(C)** TNF-α. **(D)** INF-γ. **(E)** iNOS. The data are presented as the means ± SD, *n* = 5 for each group. *P < 0.05, **P < 0.01 as compared to the control group; ^▴^*P* < 0.05, ^▴▴^*P* < 0.01 as compared to the Model group.

As shown in Figure 5, compared with the control group, the mRNA levels of IL-1β, IL-6, TNF-α, IFN-γ, and iNOS were all significantly increased in the chronic exhaustive swimming group (*P* < 0.01, *P* < 0.01, *P* < 0.01, *P* < 0.05, and *P* < 0.01, respectively). WA treatment markedly reduced the mRNA expressions of IL-1β, IL-6, TNF-α, and iNOS (*P* < 0.01, *P* < 0.01, *P* < 0.05, and *P* < 0.05, respectively) as compared to the Model group. However, the IFN-γ mRNA was not different between the Model group and the Model + WA group (*P* > 0.05).

### Effect of WA on iNOS Expression in the Hippocampus

**Figure 6:**
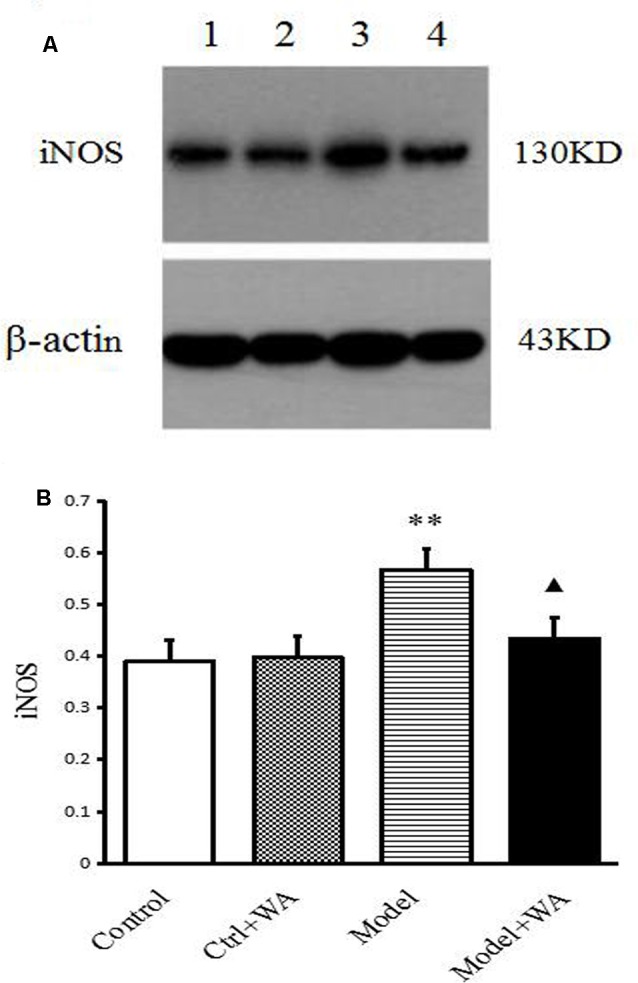
Effect of WA on iNOS expression in the hippocampus. **(A)** Representative immunoblots of iNOS and β-actin. 1. Control group; 2. Ctrl + WA group; 3. Model group; 4. Model + WA group. **(B)** Relative optical density (OD) of iNOS to β-actin. The data are presented as the means ± SD, *n* = 5 for each group. **P < 0.01; ^▴^*P* < 0.05 as compared with the Model group.

The iNOS expression in the hippocampus was measured by Western blot. As shown in Figure 6, chronic exhaustive swimming significantly increased the iNOS expression in the hippocampus (*P* < 0.01). Compared with the Model group, the iNOS of the hippocampus in the Model + WA group was significantly decreased (*P* < 0.05).

### Effect of WA on NO Content in the Serum and Hippocampus

**Figure 7:**
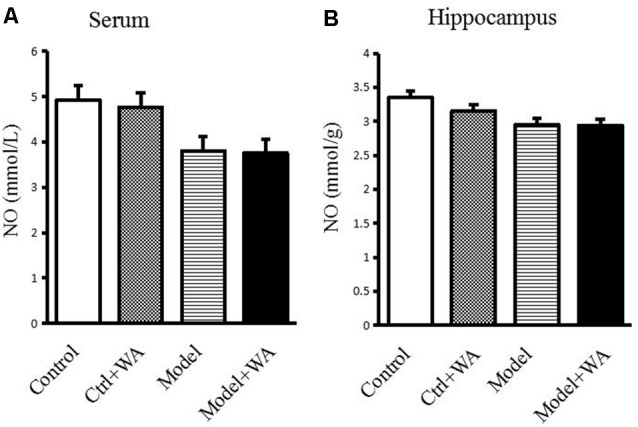
Effect of WA on NO content in the serum and the hippocampus. **(A)** NO production in the serum. **(B)** NO production in the hippocampus. The data are presented as the means ± SD, *n* = 10 for each group.

There was no significant difference in the NO production in the serum and the hippocampus among the four groups (both *P* > 0.05), as shown in Figure 7.

## Discussion

This study focused on the effects of Mongolian WA on iNOS/NO signaling pathway and proinflammatory cytokines in a chronic exhaustive swimming-induced fatigue rat model. The results showed that chronic exhaustive exercise significantly reduced the body weight and exhaustive swimming time, and induced the impairment in learning and memory, which were reversed by WA treatment. Chronic exhaustive exercise also increased the expressions of iNOS and proinflammatory cytokines. WA treatment significantly downregulated the level of iNOS and proinflammatory cytokines in chronic exercise-induced fatigue rats. These findings demonstrated that WA could alleviate the chronic exhaustive swimming-induced fatigue and improve the learning and memory ability, and the effect may be achieved by downregulating the iNOS level and reducing the expressions of proinflammatory cytokines.

Different animal models have been used in exercise-induced fatigue studies (Lu et al., 2012; Wanner et al., 2015). In this research, the model was established by chronic exhaustive swimming procedure. Animals were given adaptive swimming for 20 min in a white plastic bucket once a day for 3 days. On the fourth day, the rats began to swim with loaded weight for 21 days. We found that the body weight was significantly decreased in the exhaustive swimming group. Armstrong and VanHeest (2002) considered the loss of body weight as one of the important signs and symptoms related to overtraining and fatigue. Moreover, the exhaustive swimming time in the swimming rats was significantly shortened as compared to the original baseline. These results suggest that the chronic exercise-induced fatigue animal model was successfully established, while WA treatment could significantly increase the body weight and swimming time in exhaustive swimming rats. The results showed that Mongolian WA could improve the exercise ability of rats.

Excessive training and inadequate recovery could cause overtraining syndrome, fatigue, and tissue injury (Armstrong and VanHeest, 2002). Brain is more prone to excessive exercise injury compared to other organs (Liu et al., 2019), and it therefore might be necessary to evaluate the brain functions in excessive training. Morris water maze was a commonly used system for testing recognition, memory, and learning in animals (Zorkina et al., 2019). It can be used in combination with forced swimming protocols. The average speed, the escape latency, and the times of crossing the platform were observed in this study. We found that exhaustive swimming slightly decreased the average speed and the number of platform crossing, and mildly increased the escape latency. However, the difference was not statistically significant. Many previous studies have also reported that fatigue did not affect the learning or cognition tasks after excessive exercise (Arcelin et al., 1998). The reason might be that an easy task like that using an obvious cue (the visible flag) did not change the learning performance, but it would be impaired in the relatively difficult spatial learning task (Mizunoya et al., 2004). WA treatment could significantly increase the average speed and the number of platform crossing, and decreased the escape latency in swimming rats. The results confirmed that WA had a potent neuroprotective effect on brain damage in exhaustive exercise.

While the pathophysiology of exercise-induced fatigue remains unclear, a growing body of literature has showed that the uncoordinated activation of the immune system such as inflammation might be a causative factor (Lu et al., 2012). Appropriate exercise training induces favorable perturbations in immunity and inflammatory response, as well as a reduction in the incidence of upper respiratory tract infection (Lancaster et al., 2004). However, excessive and prolonged training with insufficient recovery might cause musculoskeletal trauma with increased production of proinflammatory cytokines such as TNF- α, IL-1β, and IL-6, which contributes to symptoms related to performance decrement and exercise-induced fatigue (Smith, 2000; Carmichael et al., 2010). Among these cytokines, IL-6 is secreted by macrophage to stimulate the immune response, e.g., fatigue and infection. It is considered to be the first cytokine present in the circulation during exercise and then initiates the cascade reaction of other cytokines and inflammatory factors. Consistent with our previous findings (Lu et al., 2012), both the mRNA expressions of TNF-α, IL-1β, and IL-6 in the hippocampus and protein levels of TNF-α, IL-1β, and IL-6 in the serum were significantly increased after chronic exhaustive exercise. As chronic fatigue in athletes suffering from overtraining may also result from peripheral proinflammatory cytokines and neuroinflammation in the brain (Smith, 2000), both peripheral and central inflammatory mechanisms were involved in the pathogenesis of exercise-induced fatigue. Our data showed that the mRNA and protein levels of TNF-α, IL-1β, and IL-6 were significantly decreased in the Model + WA group as compared to the Model group. These results suggest that WA treatment could downregulate the mRNA levels of proinflammatory cytokines in the brain and decrease the protein expressions of proinflammatory cytokines in the serum, and results in reduced inflammation response in the chronic exhaustive exercise rat.

The IFN-γ mRNA in the hippocampus and the IFN-γ concentration in the serum were also measured in this study. Our results showed that chronic exhaustive exercise significantly increased the mRNA level of IFN-γ in the hippocampus, but it did not affect the protein expression in the serum. WA treatment did not affect either the IFN-γ mRNA or IFN-γ protein. IFN-γ is a cytokine that is critical for innate and adaptive immunity against viral and intracellular bacterial infections as well as tumor control (Schoenborn and Wilson, 2007). Previous reports showed that inflammatory cytokines such as IFN-γ activate microglia in the central nervous system (Bian et al., 2008). Microglia is the mononuclear phagocytes of the brain and play a major role in brain development, synaptogenesis, and synaptic pruning (Seki et al., 2013). Microglial activation results in the production of proinflammatory cytokines (Monji et al., 2009). However, microgial cells also have an important role in neuroprotection (Upthegrove et al., 2014). Therefore, the appropriate concentration of IFN-γ in the Model + WA group might have neuroprotective effect and helps prevent overtraining injury in the central nervous system.

NO is a highly active molecule that is produced from the guanidino nitrogen of arginine by NOS. In recent years, NO has attracted attention as a potent macrophage-derived effector molecule against various bacteria and tumors (Lee et al., 2012). Under normal circumstances, iNOS expression in the brain tissue is rare, and it was increased in pathological conditions such as inflammatory reaction and immune response. Elevated expression of iNOS produces NO production, which plays a pivotal role in chronic fatigue related to nitrosative stress. We found that the protein level of iNOS in the hippocampus was significantly increased in chronic exhaustive swimming rats. WA treatment could reduce the protein expression of iNOS in fatigue rats. However, exhaustive swimming did not affect the NO production in the serum. High concentration of NO can activate macrophages to induce the production of proinflammatory cytokines IL-1β, IL-6, and TNF-α. The production of the cytokines can activate iNOS, increase NO secretion, and aggravate inflammation. Such repeated exercise stress stimulates the accelerated catabolism of L-Arg substrates, resulting in the deficiency of L-Arg *in vivo* and the reduction of NO synthesis (Song et al., 2004). Chronic exercise fatigue produces a large amount of free radicals, which can lead to the loss of biological activity of NO (Abd-ElSalam et al., 2016). Therefore, long time exercise-induced fatigue has little relation with the production of NO.

In the present study, the effects of WA in control animals were also observed. We found that WA treatment did not affect the body weight, learning ability, and inflammatory response in the sedentary control rats. However, we did not measure the exhausting time in the control group. Although these data will help to confirm the fatigue animal model, it might affect the iNOS and NO levels and proinflammatory cytokines in the control group. In addition, the exhausting time in the Model group and the Model + WA group was only measured before and at the last day of the chronic swimming period. The data in weeks 1 and 2 after chronic swimming could be useful for fatigue study, and these will be recorded in future study.

## Conclusion

Our results showed that chronic exhaustive exercise reduced the body weight and exhaustive swimming time, and induced impairment in learning and memory, which were reversed by WA treatment. Moreover, chronic exhaustive exercise resulted in the increased expressions of iNOS and proinflammatory cytokines. WA treatment significantly downregulated the level of iNOS and proinflammatory cytokines in fatigue rats. These findings demonstrated that WA could alleviate the chronic exhaustive swimming-induced fatigue and improve the learning and memory ability by reducing inflammatory response and iNOS expression in the brain.

## Data Availability Statement

All datasets generated for this study are included in the article.

## Ethics Statement

The animal study was reviewed and approved by the Institutional Animal Care and Use Committee of Affiliated Hospital of Inner Mongolia Medical University and were performed according to the ARRIVE (Animal Research: Reporting *in vivo* Experiments) guideline.

## Author Contributions

LS and R-NY: writing of the manuscript. Y-JW and L-GS: data analysis. QS and S-MB: collection and assembly of behavioral tests. G-RW and AB: supervised the overall study and advised on study design and data interpretation. Y-SC and JL: study design and data analysis.

## Conflict of Interest

The authors declare that the research was conducted in the absence of any commercial or financial relationships that could be construed as a potential conflict of interest.
